# Targeting MYC: Multidimensional regulation and therapeutic strategies in oncology

**DOI:** 10.1016/j.gendis.2024.101435

**Published:** 2024-09-16

**Authors:** Yingying Duan, Zhaoshuo Liu, Qilin Wang, Junyou Zhang, Jiaxin Liu, Ziyi Zhang, Chunyan Li

**Affiliations:** a School of Engineering Medicine, Beihang University, Beijing 100191, China; b School of Biological Science and Medical Engineering, Beihang University, Beijing 100191, China; c Key Laboratory of Big Data-Based Precision Medicine (Ministry of Industry and Information Technology), Beihang University, Beijing 100191, China; d Beijing Advanced Innovation Center for Big Data-Based Precision Medicine, Beihang University, Beijing 100191, China

**Keywords:** MYC, MYC inhibitors, MYC-Regulated biological processes, Oncogenic deregulation, Therapeutic strategies

## Abstract

MYC is dysregulated in approximately 70% of human cancers, strongly suggesting its essential function in cancer. MYC regulates many biological processes, such as cell cycle, metabolism, cellular senescence, apoptosis, angiogenesis, and immune escape. MYC plays a central role in carcinogenesis and is a key regulator of tumor development and drug resistance. Therefore, MYC is one of the most alluring therapeutic targets for developing cancer drugs. Although the search for direct inhibitors of MYC is challenging, MYC cannot simply be assumed to be undruggable. Targeting the MYC-MAX complex has been an effective method for directly targeting MYC. Alternatively, indirect targeting of MYC represents a more pragmatic therapeutic approach, mainly including inhibition of the transcriptional or translational processes of MYC, destabilization of the MYC protein, and blocking genes that are synthetically lethal with MYC overexpression. In this review, we delineate the multifaceted roles of MYC in cancer progression, highlighting a spectrum of therapeutic strategies and inhibitors for cancer therapy that target MYC, either directly or indirectly.

## Introduction

The *MYC* family consists of the oncogenes *MYC* (or *c-Myc*), *MYCN*, and *MYCL*.^1^ Although these three primary MYC family members have similar roles, their expression varies dependent on the kind of tissue and stage of development.^2^ The activity of MYC is often strictly regulated at the transcriptional, translational, and post-translational levels under physiological conditions, but up to 70% of human malignancies have abnormal expression of *MYC*, and many of these are extremely aggressive or have poor therapeutic response.^3^ As a pleiotropic transcription factor, the oncoprotein MYC controls global gene expression involved in many cellular processes, including metabolism, proliferation, senescence, cell metastasis, programmed cell death, angiogenesis, and ribosomal and protein biogenesis.^4^, ^5^, ^6^, ^7^, ^8^, ^9^, ^10^ In addition, MYC enables cancer cells to escape immune monitor through various methods.^11^ Moreover, the aberrant expression of MYC is associated with drug resistance in various tumors, emphasizing the need to target this oncogene to improve therapeutic outcomes.^12^ Due to the significant dysregulation of MYC and its direct involvement in the initiation and progression of cancer, it may be a feasible therapeutic approach to target MYC for the treatment of malignant tumors.^13^

Due to the inherent disordered structure of the MYC protein, the design of directly targeting MYC is challenging, but it is no longer deemed as an undruggable target.^14^ Many small molecules that directly target MYC-MAX have been tested in preclinical trials, and the OMO-103 cell-penetrating peptide has shown preliminary clinical efficacy in a phase I clinical trial.^15^ Alternatively, indirect targeting on MYC has been extensively explored to achieve desired anti-tumor effects.^16^^,^^17^ Studies of the key factors and mechanisms that regulate *MYC* (*e.g.*, transcription, translation, stability, and activation) will provide new opportunities to treat cancer with targeted therapy. In the present review, we classify the current therapeutic strategies for targeting MYC into four categories: i) targeting the MYC/MAX complex; ii) inhibiting the transcription or translation processes of *MYC*; iii) decreasing MYC protein stability; iv) blocking genes synthetic lethal with MYC overexpression.

## Structure of MYC protein

**Figure 1:**
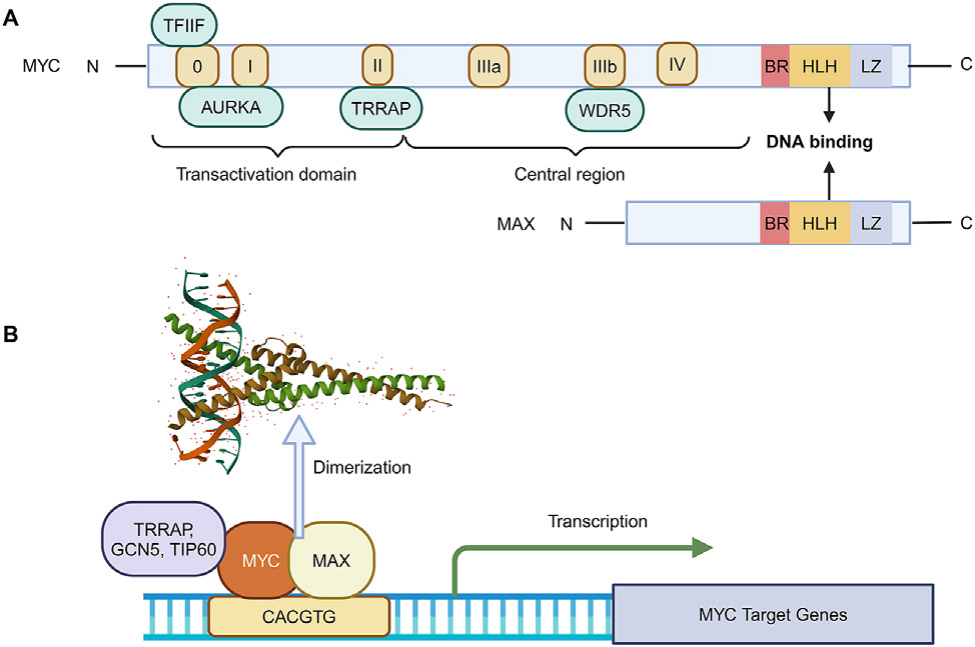
The structure and function of MYC protein. **(A)** MYC is comprised of three domains: the transactivation domain, the central region, and the DNA binding region. 0, I, II, IIIa, IIIb, and IV indicate MB0, MBI, MBII, MBIIIa, MBIIIb, and MBIV, respectively. The boxes illustrate proteins interacting with relevant MYC boxes. TFIIF, general transcription factor IIF subunit 1; AURKA, aurora kinase A; TRRAP, transformation/transcription domain associated protein; WDR5, WD repeat domain 5. **(B)** MYC and MAX combine to generate heterodimers that enhance the transcription of MYC target genes. GCN5, general control non-repressed 5 protein; TIP60 (KAT5), lysine (K) acetyltransferases 5.

The MYC protein consists of 439 amino acids and is divided into three primary domains. The N-terminal region (1–143) contains the transactivation domain, the central region is responsible for nuclear localization and stability regulation, and the C-terminal region (357–439) is crucial for interacting with MAX (MYC-associated factor X) and binding to the promoters of target genes (Fig. 1A).^18^

The MYC box (MB) refers to the highly conserved sequences of MYC proteins.^18^^,^^19^ MYC possesses six MBs, MB0, MBI, MBII, MBIIIa, MBIIIb, and MBIV (Fig. 1A). MB0, MBI, and MBII are located within the transactivation domain, while the other MBs present in the central region.^19^ MYC implements distinct functions via binding to different proteins by MB domains. MB0 enhances transcription by interacting with the TFIIF transcription elongation complex, leading to the promotion of tumor growth.^19^^,^^20^ MB0 has a critical role in MYC-induced apoptosis, regardless of the presence of p53 ^20^. MBI functions as a phosphodegron, participating in the ubiquitination and proteasomal destruction of MYC.^21^ The phosphorylation of threonine 58 (T58) and serine 62 (S62) within the MYC box I (MBI) region is crucial for controlling the activity and stability of MYC.^22^ MB0 and MBI interact with Aurora-A kinase, thereby inhibiting the binding of the ubiquitin ligase FBW7 (F-box and WD repeat domain-containing 7) to stabilize MYC protein.^23^ MBII directly interacts with TRRAP (transformation/transcription domain associated protein), a major component of the STAGA (SPT3-TAF9-GCN5 acetylase) and TIP60 (Tat-interactive protein 60 kDa) histone acetyltransferase complexes.^24^ The MBIIIa domain forms a connection with the histone deacetylase HDAC3 to suppress transcription.^25^ The MBIIIb domain interacts with WDR5 to choose its target genes based on genetic and epigenetic factors.^26^^,^^27^ Finally, MBIV is engaged in chromatin binding, facilitating the initiation of apoptosis and G2 cell arrest, hence increasing cell cycle progression.^28^^,^^29^ MBs interact with partners involved in multiple processes and pathways, such as cell proliferation and apoptosis.

The C-terminal domain, which includes the basic region (BR), helix-loop-helix (HLH), and leucine zipper (LZ), is crucial to DNA binding (Fig. 1A). Subsequently, MYC forms a heterodimer with MAX through the conserved BR-HLH-LZ motif, which facilitates the interactions between DNA and proteins.^2^^,^^30^^,^^31^ The MYC-MAX heterodimers specifically attach to the conserved E-box DNA sequence (CACGTG) within the transcription regulatory region (promoter and enhancer) of target genes. Lastly, several coactivators, such as TRRAP, GCN5 (general control non-repressed 5 protein), and TIP60/KAT5, are recruited to the E-box elements to initiate transcription activation (Fig. 1B).^2^^,^^32^

The intrinsically disordered regions (IDRs) of MYC perform in a disordered state, characterized by continuous conformational fluctuations.^33^ In the absence of MAX, MYC performs as an intrinsically disordered protein (IDP).^33^ The disorder segment of MYC predicted by PONDR® VSL2 is amino acid 146–390. The intrinsic characteristics of IDPs/IDRs have a substantial impact on the onset and formation of liquid–liquid phase separation. Consequently, it is plausible to hypothesize that MYC is engaged in liquid–liquid phase separation and transcriptional regulation.^34^

Additionally, recent research reveals that in response to proteasome inhibition, heat shock, and transcription elongation perturbation, MYC forms multimers, often in sphere-like structures.^35^ MYC multimers stabilize the connection of replication fork-associated proteins and chromatin, thereby protecting the integrity of DNA double-stranded structures and promoting tumor cell proliferation under stress conditions.^35^ Thus, the multimerization of MYC contributes to its carcinogenic properties. The inhibition of MYC multimerization will be an alternative to treat MYC-addicted tumors.

## Mechanisms on *MYC* activation in cancer

The regulation of MYC expression is precisely controlled in normal physiological settings. However, MYC is abnormally expressed in almost 70% of human malignancies.^7^, ^8^, ^9^ Numerous mechanisms, such as gene amplification, chromosomal translocation, retroviral insertion, activation of super enhancers, elevated cell signaling, and post-translational pathways, contribute to the abnormal activation of MYC in cancer cells.^36^, ^37^, ^38^, ^39^, ^40^ Genomic alterations, such as gene amplification and chromosomal translocations, can lead to increased MYC expression.^40^ Gene amplification occurs in 64% of ovarian cancer cases, 45.3% of esophageal cancer cases, 37.2% of squamous lung cancer cases, and 30% of breast cancer cases.^36^^,^^41^ The frequency of amplification of MYC, MYCL, or MYCN is 28% across 33 different forms of human cancer in The Cancer Genome Atlas (TCGA) project.^36^

**Figure 2:**
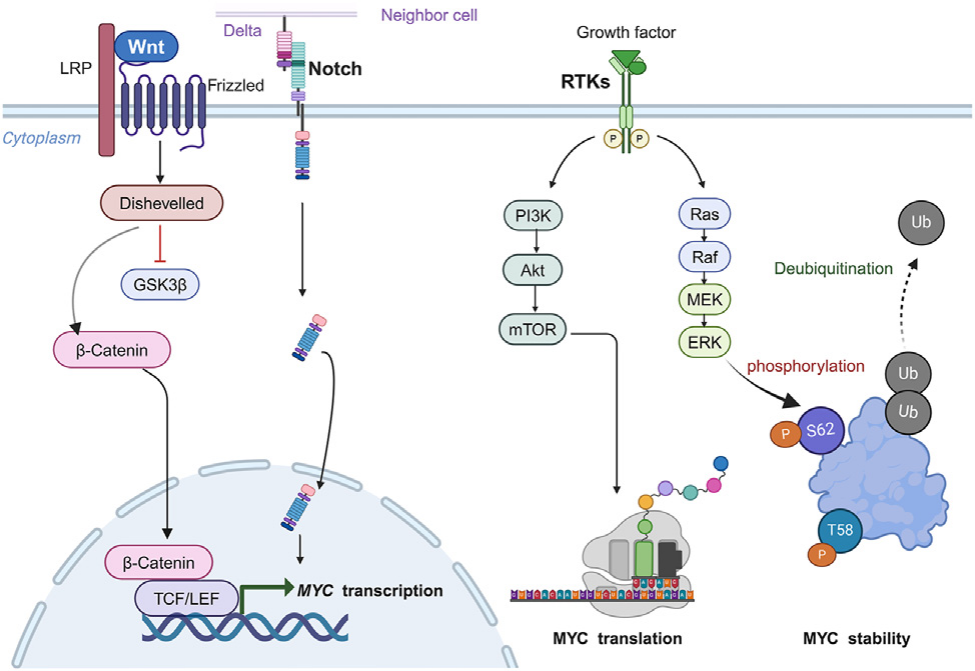
The regulation of MYC activation via multiple signaling pathways. The receptor tyrosine kinases (RTKs) influence MYC translation and protein stability through the PI3K/Akt/mTOR and Ras/ERK signaling pathways, respectively. The WNT and Notch signaling pathways control the transcription of *MYC*. T58, threonine 58; S62, serine 62; P, phosphorylation; Ub, ubiquitination; PI3K, phosphoinositide 3-kinase; ERK, extracellular signal-regulated kinase; AKT, protein kinase B; mTOR, mechanistic target of rapamycin.

In addition to the translocation or amplification, the up-regulation of *MYC* can be stimulated by multiple signaling pathways (including WNT, phosphoinositide 3-kinase/PI3K, extracellular signal-regulated kinase/ERK, Notch, *etc*.) at the mRNA or protein level (Fig. 2).^42^, ^43^, ^44^, ^45^ The activation of the WNT pathway initiates the nuclear translocation of β-catenin to form a complex with T-cell factor/lymphoid enhancer factor (TCF/LEF) to enhance the transcription of MYC.^46^ MYC is a target of the dysregulated Notch signaling pathways in T-cell leukemia.^44^ The PI3K/AKT (protein kinase B) signaling pathway enhances the nuclear translation of MYC by activating mechanistic target of rapamycin complex 1 (mTORC1) and prevents the degradation of MYC by inhibiting glycogen synthase kinase 3 beta (GSK3β).^43^ The RAS-MEK-ERK signaling pathway controls the function and durability of the MYC protein by modifying it through phosphorylation at S62.^47^ The expression of the MYC protein is minimal, and it has a transient half-life of only 30 min under normal physiological conditions.^48^ The post-translational modifications, such as phosphorylation and acetylation, increase the protein stability of MYC.^49^ On the contrary, the protein level of MYC is decreased via E3 ubiquitin ligase (*e.g.*, FBW7 or SKP2/S-phase kinase-associated protein-2) and proteasomal degradation (Fig. 2).^40^^,^^50^ However, in cancer cells, the half-life of MYC is prolonged due to abnormal post-translational modifications, resulting in excessive accumulation.^51^

## Roles of MYC in tumorigenesis

The oncoprotein MYC regulates numerous cellular processes, including proliferation, metabolism, cellular senescence, cell metastasis, ribosome and protein biosynthesis, programmed cell death, the immune response, and the tumor microenvironment.^7^^,^^12^^,^^52^, ^53^, ^54^ MYC is associated with drug resistance as well.^55^, ^56^, ^57^ In addition, MYC is involved in the biological activities and maintenance of cancer stem cells.^58^^,^^59^ The precise functions and regulatory mechanisms of MYC require further elucidation, which would be beneficial for precision medicine in cancer treatment.

## MYC and cell cycle

**Figure 3:**
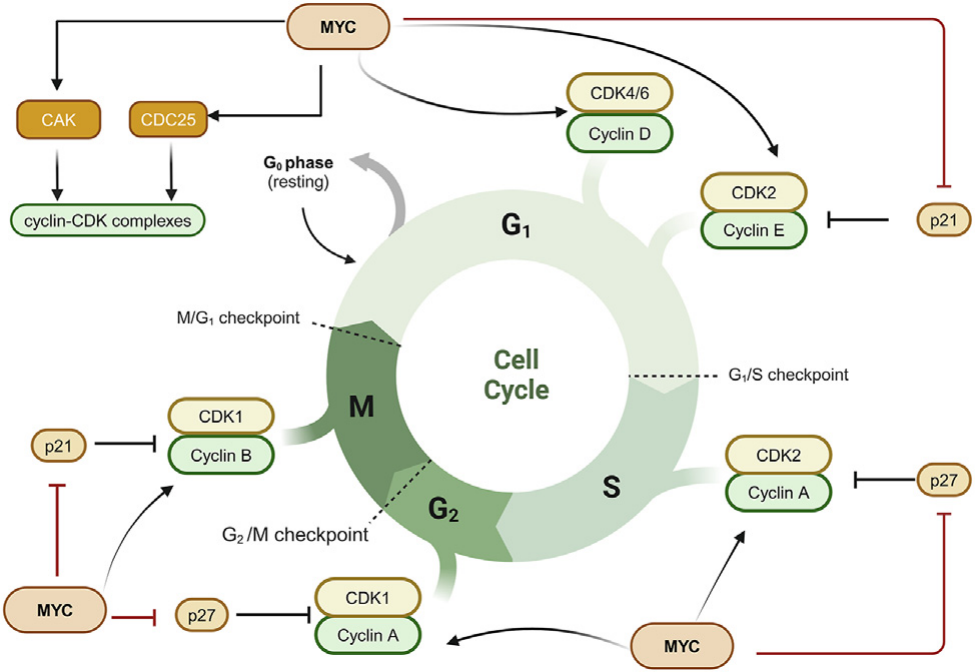
The regulation of cell cycle by MYC. The pointed arrows indicate that MYC promotes the transcription of cell cycle proteins (cyclin A, cyclin B, cyclin D, and cyclin E) and cell cycle-dependent kinases (CDK1, CDK2, CDK4, and CDK6). The red flat arrows show that MYC inhibits the activity of cell cycle inhibitors, such as p21 and p27. CAK, CDK activating kinase.

MYC enables persistent cell proliferation by forcing cancer cells to re-enter the cell cycle.^16^^,^^60^ Additionally, MYC accelerates cell cycle progression by inhibiting the cell cycle checkpoints.^61^ MYC activates the transcription of telomerase (TERT), which sustains the proliferation of tumor cells.^62^ MYC induces critical positive cell-cycle regulators, such as cyclin-dependent kinases (CDKs), cyclins (cyclin A/B/D/E), and E2F transcription factors (E2F1/2/3) (Fig. 3).^63^^,^^64^ In addition to its direct impact on transcription, MYC activates cyclin-CDK complexes by stimulating the production of CDC25 phosphatases and CDK-activating kinase (CAK) (Fig. 3).^65^

Moreover, MYC counteracts the function of CDK inhibitors, such as p21 and p27.^66^ The cell cycle inhibitors p21 (encoded by *CDKN1A*/cyclin-dependent kinase inhibitor 1A) and p27 (encoded by *CDKN1B*/cyclin-dependent kinase inhibitor 1B), play a major role in cell cycle control by blocking the kinase activity of the cyclin-CDK complex and inducing cell proliferation arrest in the G1 phase (Fig. 3).^60^ MYC represses CDKN1A transcription by binding to the zinc finger transcription factor Miz-1.^67^ MYC, Miz-1, and the DNA methyltransferase DMNT3A (DNA methyltransferase 3 alpha) form a ternary complex to repress *CDKN1A* expression by inducing CpG methylation within the promoter of *CDKN1A*.^68^ MYC induces miR-221/222 to silence p27 mRNA, resulting in the G1–S transition to promote the cell cycle.^60^

## MYC in metabolic processes

The activation of *MYC* elevates nutrient uptake, promotes glycolysis and glutaminolysis, increases fatty acid and nucleotide synthesis, and enhances oxidative phosphorylation by facilitating mitochondrial biosynthesis.^69^ MYC coordinates the metabolic processes in cancer cells to accommodate the dynamic tumor microenvironment.

## The regulation of glucose metabolism

The activation of *MYC* induces cancer cells to preferentially utilize glycolytic metabolism rather than oxidative phosphorylation, even under normoxic conditions, a phenomenon known as the Warburg effect.^70^ The aerobic glycolysis enables the tricarboxylic acid cycle intermediates to switch from ATP production to the synthesis of lipids, proteins, and nucleic acid precursors.^71^ In addition, ATP synthesis via the Warburg effect occurs at a significantly higher rate compared with oxidative phosphorylation in the presence of a sufficient glucose supply.^71^

MYC directly activates the transcription of almost all glycolytic genes, such as enolase 1 (ENO1), phosphofructokinase (PFK), phosphoglucose isomerase (GPI), hexokinase II (HK2), and lactate dehydrogenase A (LDHA), through binding to their E-box sequences.^72^ It up-regulates the glucose transporter SLC2A1 (solute carrier family 2 member 1) to enhance glucose uptake.^73^ In addition, MYC regulates lactate export by stimulating the expression of monocarboxylate transporter 1/2 (MCT1/2), hence controlling lactate levels within tumor cell.^74^ MYC stimulates the expression of glycolytic genes not only by controlling their transcription but also by influencing alternative splicing. For example, MYC maintains a high pyruvate kinase type M2 (PKM2)/pyruvate kinase type M1 (PKM1) ratio to ensure a high flux of glycolysis under aerobic conditions by stimulating the transcription of three splicing factors, namely, heterogeneous nuclear ribonucleoprotein A1/A2 (hnRNPA1/hnRNPA2) and polypyrimidine tract-binding protein (PTB).^75^ Since PKM2 facilitates the last stage of aerobic glycolysis, whereas PKM1 facilitates oxidative phosphorylation, MYC promotes glycolysis in cancer cells.^76^

## The regulation of amino acid metabolism

MYC up-regulates the expression of solute carrier family 7 member 5 (SLC7A5) and solute carrier family 43 member 1 (SLC43A1), which elevates the uptake of the essential amino acids to promote tumor progression.^77^ In addition, MYC is essential in the metabolism of glutamine, which is another nutrient for cancer cells.^78^ MYC enhances glutamine absorption by activating the glutamine transporters SLC1A5 (solute carrier family 1 member 5) and SLC38A5 (solute carrier family 38 member 5).^78^ MYC suppresses the transcription of miR-23a/b to increase the production of glutaminase 1 (GLS1), resulting in the glutaminolysis increase.^79^

## The regulation of lipid metabolism

MYC enhances the synthesis of citrate, a precursor of fatty acid in *de novo* synthesis, by increasing the activity of genes upstream of the tricarboxylic acid cycle. Additionally, MYC stimulates the transcription of fatty acid synthase (FASN), ATP citrate lyase (ACLY), acetyl-CoA carboxylase (ACC/ACACA), and stearoyl-CoA desaturase-1 (SCD1), which are involved in the process of fatty acid synthesis.^69^

## The regulation of nucleotide metabolism

MYC increases the levels of glucose-6-phosphate dehydrogenase and transketolase in the pentose phosphate pathway and subsequently enhances the production of ribose 5-phosphate.^80^ Additionally, it stimulates the production of phosphoribosyl pyrophosphate synthetase 2 (PRPS2) and phosphoribosyl pyrophosphate sequentially. Phosphoribosyl pyrophosphate acts as a framework for *de novo* purine synthesis and the salvage pathway of pyrimidine synthesis.^69^ MYC directly activates the catalytic enzymes phosphoribosyl pyrophosphate amidotransferase (PPAT) and phosphoribosyl aminoimidazole succinocarboxamide synthetase to enable nitrogen introduction in purine synthesis.^81^ During pyrimidine synthesis, MYC stimulates the production of carbamoyl-phosphate synthetase (CAD), an enzyme responsible for catalyzing the initial three processes of pyrimidine biosynthesis. In addition, MYC promotes one-carbon metabolism and the folate cycle, both of which play a role in *de novo* nucleotide synthesis.^82^

## The regulation of mitochondrial biogenesis

MYC up-regulates mitochondrial specific transcription factors and supports the structural and functional integrity of mitochondria.^83^ For instance, it up-regulates the expression of complement C1q binding protein (C1QBP), a constituent of the mitochondrial nucleoid, to promote the replication and transcription of mitochondrial DNA.^84^ In addition, AMPK-related protein kinase 5 (ARK5), a member of the AMP-activated protein kinase (AMPK) family and a target of MYC, plays a role in preserving the integrity of mitochondria and maintaining a balance in bioenergetics.^85^ SURF-1 (a respiratory complex assembly factor), TIM/TOM (inner/outer membrane transposase), and TFAM (a key regulator of mtDNA transcription and replication), as the targets of MYC, are involved in mitochondrial biogenesis as well.^12^^,^^86^

## The regulation of ribosomal and protein biogenesis

MYC directly enhances protein synthesis by up-regulating the expression of various constituents of the protein synthesis apparatus, including ribosomal proteins, translation initiation factors, RNA polymerase III, and rDNA. In addition, MYC stimulates protein biogenesis by enhancing the transcription of genes encoding translation elongation factors, translation initiation factors, nucleolar assembly components, and the small and large ribosomal subunits.^87^ For example, MYC enhances the process of modifying and processing rRNA by directly controlling ribonucleases, enzymes that change rRNA, and nucleolar proteins involved in the creation of ribosomes, such as nucleophosmin (NPM), dyskerin pseudouridine synthase 1 (DKC1), Nop52, and Nop56.^88^ Furthermore, a segment of the MYC protein is found in the nucleolus and directly regulates the production of rRNA by attaching to E-box elements found in the rDNA promoter.^87^

## The effects of MYC on the immune response and tumor microenvironment

MYC modifies the tumor microenvironment to enable cancer cells to escape from immune surveillance. Additionally, MYC stimulates the proliferation of stromal cells and induces angiogenesis.^89^ MYC is involved in the recruitment of various cytokines from the tumor microenvironment to promote the transition to a more aggressive and metastatic tumor phenotype.^89^

## MYC in angiogenesis

Vascular endothelial growth factor (VEGF) and hypoxia-inducible factor-1α (HIF-1α) are crucial cytokines in angiogenesis, and their levels are increased when MYC is activated.^90^^,^^91^ HIF-1α governs the migration of macrophages to hypoxic areas inside the tumor and controls the expression of several genes implicated in tumor angiogenesis.^90^ MYC activation leads to the up-regulation of VEGF, which leads to loss of vascular permeability and integrity, therefore, VEGF is considered the main causative factor of MYC-induced angiogenesis.^90^ MYC up-regulates the G-protein-coupled adenosine A2B receptor (ADORA2B), which promotes angiogenesis by triggering the production of VEGF and the endothelial nitric oxide synthase (eNOS).^91^ MYC activates angiogenesis by increasing the expression of interleukin (IL)-1β, as well as by inhibiting thrombospondin-1 (TSP-1), which facilitates nutrient delivery to the cancer cells.^90^

Moreover, several microRNAs regulated by MYC are implicated in angiogenesis. The activation of miR-9 in human glioma is facilitated by MYC, leading to the promotion of angiogenesis through the HIF-1α/VEGF axis.^92^ The miR-17-92 cluster effectively promotes angiogenesis by directly inhibiting the expression of anti-angiogenic molecules, such as TSP-1 and connective tissue growth factor (CTGF).^93^

## MYC and immune evasion

The immune cells infiltrated in tumor microenvironment include tumor-associated macrophages, dendritic cells, myeloid-derived suppressor cells, T cells, B cells, natural killer cells, neutrophils, *etc*.^94^ Upon the activation of *MYC*, not only more macrophages are infiltrated into the tumor, but also T, B, and natural killer cells are excluded from the tumor microenvironment.^89^

MYC promotes the expression of immune-checkpoint proteins, including cluster of differentiation 47 (CD47) and programmed cell death ligand 1 (PD-L1), to inhibit both innate and adaptive immune responses.^11^ Moreover, MYC causes cancer cells to secrete immune-inhibitory cytokines.^95^ Transforming growth factor-β (TGFβ) ligands, for instance, inhibit the function of cytolytic T cells.^95^ Furthermore, by transcriptionally suppressing signal transducer and activator of transcription 1/2 (STAT1 and STAT2) and the type I interferon pathway, MYC impairs natural killer cell-mediated immune surveillance.^96^ In addition, MYC increases the expression of many cytokines, including IL-23 and C–C motif chemokine ligand 2/9 (CCL2 and CCL9), which promote the recruitment of immunosuppressive macrophages and decrease the recruitment and activation of natural killer cells, B cells, and CD4^+^/CD8^+^ T cells.^97^

## MYC and senescence

The suppression of *MYC* induces cellular senescence in different types of cancer cells, including osteosarcoma and hepatocellular carcinoma.^98^ MYC enables cancer cells to resist senescence by being dependent on cell cycle protein-dependent kinase 2 (CDK2).^99^ MYC inhibits senescence by activating long noncoding RNA (lncRNA) USP2-AS1 to stabilize *E2F1* mRNA.^100^

## MYC and cell metastasis

Metastasis requires the coordination of various molecules involved in invasion, chemotaxis, and contractile activity.^101^ MYC overexpression leads to the enhanced transcription of ezrin to induce the up-regulation of downstream genes, such as Akt and Ras homolog family member A (RhoA), which play an important role in cell invasion.^102^

MYC-nick is a truncated form of full-length MYC cleaved by the endogenous proteasome.^103^ MYC-nick enhances the production of fascin (an actin-bundling protein) and stimulates the activation of cell division cycle 42 (Cdc42) to rearrange the actin cytoskeleton to form filopodia, facilitating cell migration.^103^ In addition, MYC promotes cell migration by increasing the expression of genes associated with epithelial-to-mesenchymal transition, such as galectin 1 (LGALS1), osteopontin (OPN), and SNAIL.^12^^,^^104^ However, a study in *Drosophila* and lung adenocarcinoma cell lines discovered that MYC impedes migration and invasion mediated by Ras and lethal giant larvae (Lgl).^105^ The binding between urokinase (uPA) and urokinase receptor (uPAR) initiates the reorganization of cytoskeletal structure to impede cells' invasion into the extracellular matrix.^106^ MYC suppresses the expression of uPA and uPAR in cancer cells to stimulate metastasis.^107^

## MYC and programmed cell death mechanisms

MYC is involved in various types of programmed cell death, including apoptosis, autophagy, pyroptosis, and ferroptosis. MYC regulates cell apoptosis through two main pathways: BCL-2 (B-cell lymphoma 2) pathway and p53 pathway. MYC inhibits BCL-2 and BCL-XL (BCL2 like 1) by promoting the transcription of BIM (BCL-2–interacting mediator of cell death), ultimately inducing apoptosis.^108^^,^^109^ The downstream activation of p53 is important for the apoptotic induction by MYC as well.^108^ The activation of MYC results in the overexpression of ADP-ribosylation factor (ARF), which enhances the levels of p53 to trigger apoptosis.^12^ MYC inhibition leads to defects in autophagosome formation and reduced delivery of autophagic substrates.^110^ Knockdown of MYC down-regulates the transcription of autophagy-related protein 7 (Atg7) and LC3-I (a cytosolic form of 1A/1B-light chain 3)/LC3-II (LC3-phosphatidylethanolamine conjugate), resulting in autolysosomal degradation.^111^ In multiple myeloma cells, the transcriptional inhibition on *MYC* results in caspase-1-dependent pyroptosis.^112^ By blocking nuclear receptor coactivator 4 (NCOA4)-mediated ferritin autophagy, MYC prevents ovarian cancer cells from ferroptosis.^112^ MYC also activates the expression of lymphoid-specific helicase, which inhibits ferroptosis.^113^

## MYC and drug resistance

The expression of ATP-binding cassette (ABC) transporters, especially the P-glycoprotein (P-gp), can cause resistance to targeted chemotherapy and cytotoxic.^114^ MYC overexpression leads to the activation of several intermediate factors, such as small nucleolar RNA host gene 12 (SNHG12), HIF-1α, nuclear factor erythroid 2-related factor 2 (Nrf2), and miR-20a, which increase the levels of P-glycoprotein (P-gp) and promote the development of multidrug resistance.^55^, ^56^, ^57^ MYC also interacts with bromodomain PHD finger transcription factor (BPTF) to increase the expression of ABC transporter, resulting in multidrug resistance.^115^

## The therapeutic exploration of MYC

Historically, most of the scientific community believed that MYC was essentially “undruggable”. Due to its intrinsically disordered regions, lacking binding pockets, and its sequestration within the nuclear compartment, it is challenging to explore MYC as a therapeutic target.^116^ Recently, the covalent ligand targeting of intrinsically disordered proteins and the antibody delivery system to the nucleus have been developed.^117^ In the absence of conventional binding pockets, drugs can bind to MYC proteins via cysteine-responsive covalent ligands.^117^^,^^118^ In 2023, scientists developed biodegradable silica nanocapsules to transfer antibodies directly to the nucleus for cancer therapy.^119^ This nuclear delivery system has the potential to be applied to the MYC targeting to cure cancer.

Although targeting MYC is challenging, it cannot simply be assumed that MYC is “undruggable”.^14^ Currently, effective approaches to MYC targeting include interfering with the MYC-MAX complex, blocking MYC transcription and translation, promoting MYC proteasomal degradation, and utilizing synthetic lethality. Next, we outline various approaches to targeting MYC, along with recent advances in MYC inhibitors (Table 1).

**Table 1:** Summary on MYC targeting agents and the mechanisms of action (MOA).

Strategy	MOA	Agents	Malignancy	Preclinical/Clinical stage	References
Targeting the MYC-MAX complex directly	Blocking MYC-MAX interaction	Omomyc (OMO-103)	Advanced solid tumors	Phase I/II: NCT04808362	15
		3jc48-3	Patient-derived prostate cancer xenografts (PDX) mouse models	Preclinical	121
		KJ-Pyr-9	MDA-MB-231 breast cancer CDX model	Preclinical	123
		MYCMI-6	Neuroblastoma xenograft tumor model	Preclinical	124
		MYCi361 and MYCi975	MyC-CaP allograft/xenograft mouse prostate model	Preclinical	122
		EN4	MDA-MB-231 breast cancer CDX model	Preclinical	118
	Stabilizing MAX-MAX homodimers	KI-MS2-008	T-cell acute lymphoblastic leukemia and hepatocellular carcinoma mouse models.	Preclinical	125
	Blocking MYC-MAX from binding to DNA	KSI-3716	Orthotopic bladder xenografts	Preclinical	127
		ME47	Breast cancer CDX model	Preclinical	126
Silencing the transcription of MYC	Stabilizing G-quadruplex DNA	CX-5461	Solid tumors	Phase I: NCT02719977	130
		IZCZ-3	SiHa, HeLa, Huh7, A375 cell lines and human cervical squamous	Preclinical	131
		QN-1	Triple-negative breast cancer (TNBC) mouse model	Preclinical	132
		IZTZ-1	Breast cancer xenograft mouse model	Preclinical	133
	BRD4 inhibitors	ZEN-3694	Metastatic castration-resistant prostate cancer (mCRPC)	Phase I/II: NCT02711956	137
		OTX015/MK-8628	Advanced solid tumors	Phase I: NCT02698176 Phase II: NCT02296476	140
		AZD5153	Malignant solid tumors, lymphoma and breast cancer	Phase I: NCT03205176	138,139
		GSK525762/I-BET762	Neoplasms	Phase I/II: NCT01943851	141
	CDK7 inhibitors	CT7001 (samuraciclib)	Advanced solid malignancies	Phase I: NCT03363893	146
		LY3405105	Solid tumors	Phase I/II: NCT03770494	148
		SY-1365	Advanced solid tumors	Phase I: NCT03134638	149
		SY-5609	Advanced solid tumors	Phase I: NCT04247126	150
	CDK9 inhibitors	KB-0742	Relapsed or refractory solid tumors	Phase I/II：NCT04718675	151
		CYC065	Advanced cancers	Phase I: NCT02552953	153
		SCH 727965 (Dinaciclib)	Advanced breast and lung Cancers	Phase II: NCT00732810	154
Inhibiting the MYC protein biosynthesis	mTOR inhibitors	MLN0128	Metastatic castration-resistant prostate cancer	Phase II: NCT02091531	156
		Rapamycin	Lymphangioleiomyomatosis	clinical use	155
		Temsirolimus and everolimus	Advanced renal cell carcinoma (RCC)	clinical use	155
	eIF4A inhibitor	eFT226	Solid tumors	Phase I-II: NCT04092673	158
Decreasing the MYC stability	USP7 inhibitors	XL177A	Ewing sarcoma and malignant rhabdoid tumor (MRT) cell lines	Preclinical	162
		GNE-6640, GNE-6776	EOL-1 xenograft models	Preclinical	163
		FT671	Colorectal carcinoma (HCT116) or bone osteosarcoma (U2OS) cell lines	Preclinical	164
	USP36 inhibitor	Cinobufotalin	Colon cancer cell lines and xenograft models	Preclinical	165
	PP2A activators	SMAP	KRAS-driven non-small cell lung cancer and triple-negative breast cancer xenograft models	Preclinical	168,169
	PLK1 inhibitors	BI6727	NSCLC	Phase II: NCT00824408	171
		BI2356	Neoplasms	Phase I: NCT02211872	172
		NMS-1286937	Advanced solid tumors	Phase I: NCT01014429	173
		GSK461364	Advanced solid tumors or lymphoma	Phase I: NCT00536835	174
	PIN1 inhibitor	Sulfopin	Murine models of neuroblastoma and pancreatic cancer	Preclinical	177
	PROTAC	PROTAC targeting MYC	TNBC cells	Preclinical	179
		ProMyc	Xenograft tumor models	Preclinical	180
Targeting synthetic-lethal genes with MYC	CDK1 inhibitor	RO-3306	Ovarian cancer cells and a transgenic mouse model of ovarian cancer	Preclinical	184
	CHK1 inhibitors	CBP501	Advanced solid tumors	Phase I: NCT03113188	187
		LY2603618	Non-small cell lung cancer	Phase II: NCT00988858	188
		AR323 and AR678	Melanoma cell lines	Preclinical	189
	PRKDC inhibitor	Peposertib (M3814)	Advanced solid tumors or chronic lymphocytic leukemia	Phase I: NCT02316197	190
	ATR inhibitor	Ceralasertib (AZD6738)	Advanced solid tumors	Phase I: NCT04497116	191
	IMPDH2 inhibitor	AVN-944	Refractory solid tumors	Phase I: NCT00923728	192

## Targeting the MYC-MAX complex

**Figure 4:**
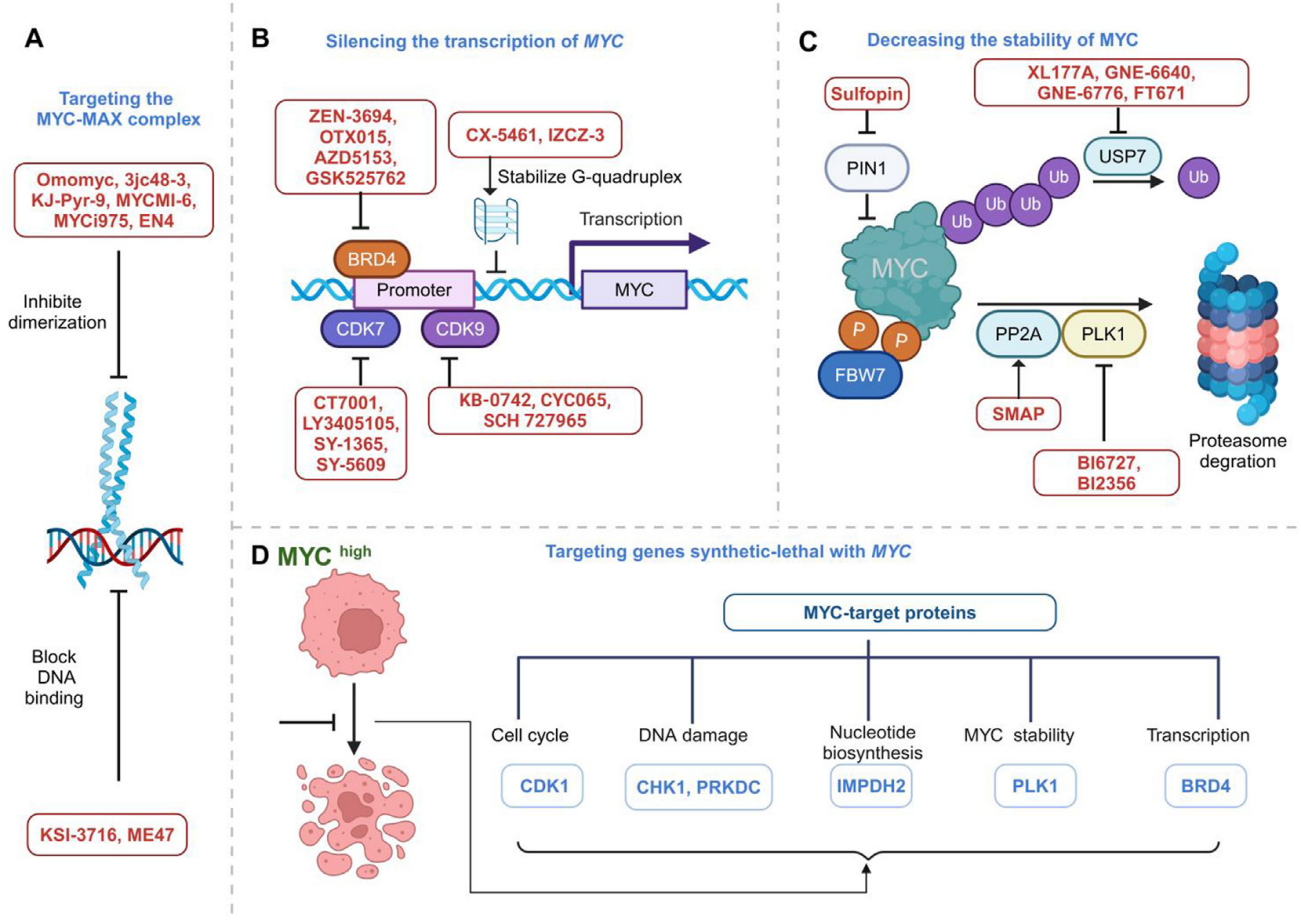
The therapeutic strategies for targeting MYC. **(A)** Small compounds directly suppress MYC by preventing MYC-MAX from dimerizing or by preventing MYC-MAX from binding to specific DNA regions. **(B)** Indirect targeting of MYC by silencing the transcription of MYC. **(C)** Indirect targeting of MYC by decreasing the stability of MYC. **(D)** Inhibiting synthetic lethal genes with MYC. BRD4, bromodomain-containing protein 4; PP2A, protein phosphatase 2A; PLK1, polo-like kinase-1; CDK, cyclin-dependent kinase; CHK1, checkpoint kinase 1; IMPDH, inosine-5-monophosphate dehydrogenase; GLS, glutaminase; Me, methylation; P, phosphorylation; Ub, ubiquitylation.

The MYC-MAX complex binds to DNA to activate the target gene's transcription.^2^ To inhibit MYC signaling, small molecules have been developed to target the interface between MYC and MAX. These molecules can stabilize the MAX-MAX homodimer or disrupt the binding of MYC-MAX to DNA (Fig. 4A). The only direct inhibitor of MYC in clinical phase I/II trials is Omomyc (OMO-103), which heterodimers with MAX to prevent transcription of MYC targets.^120^ Recently, results from a dose-escalation phase I study in solid tumors showed initial indications of the safety and the drug activity of OMO-103.^15^ 3jc48-3 was well tolerated and effective in reducing tumor growth rates in mouse models.^121^ The MYC inhibitors 361 and 975 (MYCi361 and MYCi975) exhibit substantial anti-tumor effectiveness by disrupting the MYC-MAX and promoting MYC phosphorylation on threonine-58.^122^ Compared with MYCi361, MYCi975 showed increased tolerance at significantly higher doses.^122^ KJ-Pyr-9 and MYCMI-6, small molecules inhibiting MYC-MAX interactions, induce massive apoptosis and reduce proliferation in xenograft mouse models with MYC-driven tumors.^123^^,^^124^ EN4, which was found through a cysteine-responsive covalent ligand screen, targets cysteine 171 (C171) within the intrinsically disordered region of MYC.^118^ In the MDA-MB-231 breast cancer cell line-derived xenograft model, EN4 decreased MYC-MAX DNA binding, resulting in compromised cell proliferation and tumor growth *in vivo*.^118^ The compound KI-MS2-008 stabilizes the MAX–MAX homodimers to decrease the expression of MYC target genes and decreases tumor growth *in vivo*.^125^

Unlike small compounds that impede heterodimerization, compound KSI-3716 and ME47 specifically prevent the binding of MYC-MAX to DNA.^126^^,^^127^ In a cell line-derived xenograft model of breast cancer, ME47 decreases cell proliferation, slows tumor growth, and increases survival.^126^ In murine orthotopic bladder xenografts, KSI-3716 suppresses tumor growth without significant systemic toxicity.^127^

## Silencing the transcription of *MYC*

The transcription of *MYC* can be inhibited in two ways: the first is to stabilize the MYC G-quadruplex structure, and the second is to target the transcriptional complex (Fig. 4B). The G-quadruplex structure is a noncanonical DNA structure near the promoter region of oncogenes, such as *MYC*. The three-dimensional structure of G-quadruplex provides a natural binding pocket for small molecules to inhibit transcription.^128^ G-quadruplexes regulate gene expression, including 90% of MYC expression, thereby making them potential drug targets for *MYC* addictive cancers.^128^ Small molecules and peptides have been identified to bind to and stabilize the MYC G4 structure, such as CX-5461, IZCZ-3, QN-1, and IZTZ-1.^129^ For the treatment of solid cancers, CX-5461 was well tolerated in a phase I clinical trial.^130^ The three remaining inhibitors showed significant anti-tumor effects in both cancer cell lines and mouse models.^131^, ^132^, ^133^

Super-enhancers (SEs) are clusters of enhancers densely occupied by transcription factors and chromatin regulators.^134^ In addition to SEs, the transcription of *MYC* is regulated by a variety of transcriptional complexes, such as CDKs, bromodomain and extra-terminal (BETs) domains, and RNA polymerase II.^135^ Bromodomain-containing protein 4 (BRD4), a member of BETs, plays a major role in *MYC* transcription.^136^ BRD4 stimulates the transcriptional activation and elongation of MYC via the recruitment of positive transcription elongation factor b (P-TEFb).^136^ Several BET inhibitors are currently being evaluated in phase I or phase II clinical trials for cancer treatment, including ZEN-3694, OTX015/MK-8628, AZD5153, and GSK525762/I-BET762.^137^, ^138^, ^139^, ^140^, ^141^ Due to the limited therapeutic activity of BET inhibitors, it is alternative to combine with other epigenetic reagents.^142^

Unlike the traditional cell cycle CDKs mainly involved in cell cycle transition, CDK7 and CDK9 play crucial roles in the initiation and elongation of transcription.^143^^,^^144^ CDK7 functions as the catalytic component of the transcription factor IIH complex (TFIIH), while CDK9 serves as the kinase component of P-TEFb.^143^^,^^144^ CDK7 and CDK9 phosphorylate the serine residues on the carboxy-terminal of RNA polymerase II, which helps to initiate transcription, release pauses, and facilitate elongation efficiently.^145^ The CDK7 inhibitor, CT7001, has undergone clinical phase I trials in advanced breast cancer with an acceptable safety profile and preliminary evidence of efficacy.^146^ In addition, the CDK7 inhibitors LY3405105, SY-1365, and SY-5609 have entered phase I/II clinical trials in solid tumors.^147^, ^148^, ^149^, ^150^ CDK9 inhibitors, such as KB-0742, CYC065, and SCH 727965 (dinaciclib) have demonstrated anti-tumor activity *in vivo* and are currently being investigated in phase I/II clinical trials.^151^, ^152^, ^153^, ^154^

## Inhibition of the MYC protein biosynthesis

Under typical physiological circumstances, mTORC1 phosphorylates eukaryotic initiation factor 4E (eIF4E)-binding protein 1 (4EBP1) to release the eukaryotic initiation factor 4G (eIF4G)-binding sites in eIF4E and start the translation of MYC. Rapamycin, a pharmaceutical inhibitor of mTORC1, decreases the expression of MYC and has been in clinical use.^155^ Rapamycin derivatives, temsirolimus and everolimus, retain the skeletal structure of rapamycin but have improved solubility and pharmacokinetic properties.^155^ MLN0128 (sapanisertib), a novel mTOR inhibitor as the next generation of rapamycin and its analogues, has been evaluated in phase II clinical trials, but its efficacy is limited.^156^

The helicase activity of eukaryotic initiation factor 4A (eIF4A) is crucial for overcoming structural barriers caused by G-quadruplexes, therefore ensuring the continuous translation of MYC mRNA.^157^ eFT226, an inhibitor of eIF4A helicase, effectively reduces MYC protein levels without affecting MYC mRNA levels and is in a phase I-II clinical trial.^158^ This study has shown that eFT226 is effective in advanced solid tumors.

## Decrease in MYC stability

The stability of MYC is controlled by phosphorylation and ubiquitination. Phosphorylation of Ser62 enhances the stability of MYC, while phosphorylation of Thr58 leads to its destruction.^49^ The MYC protein is tightly regulated by the ubiquitin-proteasome system, leading to a relatively short half-life of approximately 30 min under normal physiological conditions. MYC proteins are degraded by the ubiquitin-proteasome system in two stages. First, poly-ubiquitin is covalently tagged onto the target protein. Second, the 26S proteasome recognizes and accepts the tagged protein with the right ubiquitin linkages. Then, the tagged protein is deubiquitinated, unfolded, and degraded into tiny peptide fragments by the proteasome.^49^

One strategy for MYC degradation is to target deubiquinating enzymes (DUBs).^159^ DUBs, including USP7, USP13, USP22, USP28, USP36, USP37, and USP43, deubiquitinate and stabilize MYC family proteins.^160^^,^^161^ The inhibition of these DUBs increases the proteasomal degradation of MYC and reduces the transcription of MYC-driven genes.^160^ The inhibitors targeting the above DUBs are currently in preclinical studies. USP7 inhibitors, including GNE6776, XL177A, FT671, and GNE-6640, effectively hinder the proliferation of many cancer cell lines and impede tumor growth in xenograft models.^162^, ^163^, ^164^ The newly discovered USP36 inhibitor, cinobufotalin, suppresses the malignant phenotypes of colon cancer cells both *in vitro* and *in vivo*.^165^ For the remaining five DUBs, no specific inhibitors have yet been reported.

T58 undergoes phosphorylation by GSK3β as a result of a complicated signaling cascade.^49^ The cascade is triggered by mitogen-activated protein kinases (MAPKs) and CDKs, which cause the addition of a phosphate group to serine 62 (S62) on MYC. GSK3β phosphorylates T58 after the phosphorylation of S62. However, the dephosphorylation of S62 by the protein phosphatase PP2A (protein phosphatase 2A) is necessary to recognize MYC by F-box and WD repeat domain-containing 7 (FBW7).^49^^,^^166^ Several methods have been explored to improve the degradation of MYC, including the inhibition of PI3K to promote the action of GSK3β and using small-molecule activators of PP2A (Fig. 4C).^167^ SMAP (small-molecule activators of PP2A) treatment inhibited tumor growth in xenograft models of KRAS-driven non-small cell lung cancer and triple-negative breast cancer.^168^^,^^169^

By directly binding and phosphorylation, Polo-like kinase-1 (PLK1) promotes FBW7's polyubiquitination and destruction, resulting in the stabilization of MYC (Fig. 4C).^170^ There have been more than a dozen available PLK1-specific inhibitors, of which BI6727, BI2356, NMS-1286937, and GSK461364, have entered clinical trials in solid tumors. Although they have acceptable safety profiles, their efficacy is limited and they may induce drug resistance.^171^, ^172^, ^173^, ^174^, ^175^ Additionally, PIN1 is a member of the peptidylprolyl cis/trans isomerase (PPIase) family and increases the stability and transcriptional activity of MYC (Fig. 4C).^176^ Sulfopin, a covalent inhibitor of PIN1, reduces tumor progression in mouse models of neuroblastoma and pancreatic cancer.^177^

As a novel drug design strategy, proteolysis targeting chimera (PROTAC) is being explored to cure cancer.^178^ A PROTAC based on TNA (threose nucleic acid) and DNA has been developed to effectively target and degrade MYC in triple-negative breast cancer cells, which provides a promising therapeutic intervention for triple-negative breast cancer.^179^ In addition, a recent study identified a specific anti-MYC aptamer MA9C1 and further developed a multifunctional aptamer-based PROTAC for the proteolysis of MYC (ProMyc).^180^ ProMyc not only significantly degrades MYC, but also reduces MAX proteins subsequently.^180^ The circular PA1-ProMyc chimeras achieved tumor regression in xenograft tumor models, laying the foundation for the clinical development of effective MYC degraders.^180^

## Targeting genes synthetic lethal with MYC overexpression

Synthetic lethal interactions occur between two genes when the individual perturbation of either gene makes the cell/organism viable, but the simultaneous perturbation of both genes results in lethality.^181^ Synthetic lethality has the potential to selectively target oncogenic MYC to minimize harm to normal cells.

The crucial factor in harnessing synthetic lethal interactions for cancer therapy lies in the discovery of synthetic lethal genes (Fig. 4D). Large-scale genetic screens have explored noncanonical sensitivities in MYC-driven cancers, and the range of potential synthetic lethal targets is expanded. With the ongoing advancement of high-throughput screening technologies, over a hundred candidate genes could be synthetically lethal with the dysregulated oncoprotein MYC.^182^

Potential synthetic lethal targets are genes that function together with MYC to promote cell cycle progression or metabolism. CDK1 is a synthetic lethal target of MYC in cancer cells.^183^ CDK1 inhibitor RO-3306 has anti-tumor effects in ovarian cancer cells and mouse xenograft models implanted with ovarian cancer cells.^184^ MYC has been found to target DNA damage checkpoint regulators, such as checkpoint kinase 1 (CHK1), TPX2 (microtubule nucleation factor), catalytic subunit (PRKDC), and ataxia telangiectasia and Rad3-related protein (ATR), as synthetic lethal targets to counteract the effects of increased proliferation-related elevated mitotic stress and DNA damage.^185^^,^^186^ CBP501 and LY2603618, the CHK1 inhibitors, had acceptable safety and pharmacokinetic profiles in clinical phase I trials, but they did not present promising anti-cancer effects in clinical phase II trials.^187^^,^^188^ More CHK1 inhibitors, such as AR323 and AR678, are still in preclinical development.^189^ The PRKDC inhibitor peposertib and ATR inhibitor ceralastetib were well tolerated in phase I clinical trials.^190^^,^^191^

Since MYC-driven tumors heavily depend on glucose and glutamine, the simultaneous suppression of both glycolysis and glutaminolysis could potentially provide a promising synergistic treatment for MYC-driven malignancies. Enhanced production of nucleic acid and protein is required to sustain MYC-induced cellular proliferation. Inosine-5-monophosphate dehydrogenase 2 (IMPDH2) is a crucial enzyme involved in nucleotide biosynthesis and is identified as a synthetic lethal target of MYC.^182^ The inhibitor of IMPDH2, AVN-944, is currently in a phase I clinical trial, under the evaluation of safety and efficacy.^192^ Numerous genes described above that regulate MYC expression and stability have also been identified as synthetic lethal targets for MYC-driven tumors, including PLK1 and BRD4.^182^ Studies on inhibitors of PLK1 and BRD4 have been described previously.

The development of medications that target MYC has made significant strides. However, due to the extensive influence of MYC on cells, it is crucial to assess the side effects of MYC inhibition in cancer treatment. The mice with MYC knockout exhibit early aging and their age-sensitive abilities decline, but they also live noticeably longer than wild-type mice and have a 3-to-4-fold lower cancer incidence.^193^ MYC knockout resulted in electron transport chain dysfunction and increased production of reactive oxygen species.^193^ The mitochondrial abnormalities may potentially accelerate aging in MYC knockout mice.^193^ Therefore, we need to be mindful of the possible adverse effects of premature aging and mitochondrial dysfunction, particularly when MYC inhibitors are applied in children's cancers.

## Conclusions

MYC is a potential therapeutic target for cancer since it controls both the immune system's responses and the intrinsic development of tumor cells. Cancer growth is slowed in a number of preclinical cancer models when MYC expression or activity is removed. We outline many approaches to cancer treatment that target MYC both directly and indirectly. Nevertheless, several challenges still exist in the therapeutic exploration of MYC. First, the development and optimization of direct MYC inhibitors are still hampered by the intricate structure of the MYC protein. Recent developments in protein structure prediction could be a significant step forward in the improvement of imperfect crystallographic data. However, most research institutions may find it difficult to meet the increasing demand for computer resources, and no model can now fully understand the intricacy of intrinsically disordered domains. Secondly, it is both chemically and pharmacologically feasible to suppress MYC expression by targeting the regulatory factors. However, a lot of other genes or proteins are targeted simultaneously as well, and this pleiotropic effect makes it difficult to find drugs with minimal side effects. Targeting the stability of the MYC protein with selectivity is also difficult. Therefore, it is debatable if these techniques' ability to selectively target MYC is what makes them therapeutically effective. Third, due to MYC’s interactions with so many distinct proteins, which are often context-dependent, the effects of indirect MYC inhibition are probably specific to certain tumor types. Ultimately, given MYC's pervasive role in cancer, it is crucial to contemplate the potential adverse effects of MYC inhibition, such as premature aging.

Combination therapies are commonly used to enhance the efficacy of cancer treatment, as resistance to single therapies has become a significant issue. Emerging treatments that target MYC, its downstream genes, or synthetic lethal partners could potentially be integrated into future clinical strategies for patient stratification and the selection of combination therapy regimens.

## Funding

This work was supported by grants from the 10.13039/100014717National Natural Science Foundation of China (No. 32270610, 82072499, 31801094 to Chunyan Li) and the Fundamental Research Funds for the Central Universities (China) (No. YWF-21-BJ-J-T105 to Chunyan Li).

## CRediT authorship contribution statement

**Yingying Duan:** Investigation, Visualization, Writing – original draft, Writing – review & editing. **Zhaoshuo Liu:** Visualization. **Qilin Wang:** Investigation, Writing – review & editing. **Junyou Zhang:** Investigation, Writing – review & editing. **Jiaxin Liu:** Writing – review & editing. **Ziyi Zhang:** Writing – review & editing. **Chunyan Li:** Conceptualization, Funding acquisition, Writing – review & editing.

## Declaration of Generative AI and AI-assisted technologies in the writing process

During the preparation of this work, the authors used Kimi to improve the writing and readability of some sentences in the manuscript. After using these tools, the authors reviewed and edited the content as needed. The authors take full responsibility for the content of the publication.

## Conflict of interests

The authors declared no conflict of interests.
